# Caveolin 1 Promotes Renal Water and Salt Reabsorption

**DOI:** 10.1038/s41598-017-19071-6

**Published:** 2018-01-11

**Authors:** Yan Willière, Aljona Borschewski, Andreas Patzak, Tatiana Nikitina, Carsten Dittmayer, Anna L. Daigeler, Markus Schuelke, Sebastian Bachmann, Kerim Mutig

**Affiliations:** 1 Department of Anatomy, Charité-Universitätsmedizin Berlin, Berlin, Germany; 2 Department of Physiology, Charité-Universitätsmedizin Berlin, Berlin, Germany; 3 Department of Neuropediatrics, Charité-Universitätsmedizin Berlin, Berlin, Germany

## Abstract

Caveolin-1 (Cav1) is essential for the formation of caveolae. Little is known about their functional role in the kidney. We tested the hypothesis that caveolae modulate renal salt and water reabsorption. Wild-type (WT) and Cav1-deficient (Cav1−/−) mice were studied. Cav1 expression and caveolae formation were present in vascular cells, late distal convoluted tubule and principal connecting tubule and collecting duct cells of WT but not Cav1−/− kidneys. Urinary sodium excretion was increased by 94% and urine flow by 126% in Cav1−/− mice (p < 0.05). A decrease in activating phosphorylation of the Na-Cl cotransporter (NCC) of the distal convoluted tubule was recorded in Cav1−/− compared to WT kidneys (−40%; p < 0.05). Isolated intrarenal arteries from Cav1−/− mice revealed a fourfold reduction in sensitivity to phenylephrine (p < 0.05). A significantly diminished maximal contractile response (−13%; p < 0.05) was suggestive of enhanced nitric oxide (NO) availability. In line with this, the abundance of endothelial NO synthase (eNOS) was increased in Cav1−/− kidneys +213%; p < 0.05) and cultured caveolae-deprived cells showed intracellular accumulation of eNOS, compared to caveolae-intact controls. Our results suggest that renal caveolae help to conserve water and electrolytes via modulation of NCC function and regulation of vascular eNOS.

## Introduction

Caveolae are flask-like, 60 to 80 nm-size, cholesterol- and sphingolipid-enriched invaginations of the plasma membrane. They are typically found in endothelial and smooth muscle cells as well as in some epithelia^1,2^. Previous work has demonstrated their ability to provide plasma membrane reservoirs during mechanical stress such as osmotic swelling or axial stretching^3^. Apart from this role, caveolae have been implicated in multiple cell functions such as signal transduction, vesicular trafficking, endocytosis, and functional modulation of plasma membrane proteins^1,4^. Major pathways such as nitric oxide release or calcium signaling have been associated with caveolae^1,4^. Caveolae have been implicated in regulation of vascular tone, cardiac rhythm, respiratory function, and overall lipid metabolism^5–7^.

Caveolin-1 (Cav1) and Cavin-1 (also known as Polymerase I and Transcript Release Factor; PTRF) are essential for the biogenesis of caveolae. Genetic deletion of either Cav1 or PTRF in mice leads to impaired caveolae formation with resulting functional disorders primarily affecting blood vessels, lungs, and fat tissue^5,6,8^. Human PTRF mutations have been linked with congenital generalized lipodystrophy type 4 (CGL4) characterized by markedly reduced body fat mass, muscle weakness, and life-threatening cardiac arrhythmia^7^. Although caveolae are abundant in virtually all organs, previous studies were mainly focused on their functional relevance in the respiratory and cardiovascular systems^9^. Caveolae have been implicated in the pathogenesis of pulmonary diseases such as asthma, obstructive disease, and fibrosis, as well as cardiovascular disease including pulmonary hypertension^10^. Less is known about the role of caveolae in the kidney, where earlier studies described the presence of Cav1 and caveolae in the vasculature and distal renal epithelia^11^. Phenotyping of Cav1-deficient mice (Cav1−/−) revealed moderate urinary loss of calcium, magnesium, and potassium, suggesting that caveolae may play a role in renal handling of these electrolytes^12,13^. These effects are believed to depend on functional interactions of Cav1 with basolateral calcium and potassium transport proteins^12,13^. A recent study in vasopressin-deficient Brattleboro rats with central diabetes insipidus (DI) proposed a role for Cav1 in the urinary concentration process; stimulation of DI rats with the vasopressin V2 receptor agonist desmopressin (dDAVP) induced a sustained apical translocation of Cav1 in principal cells of collecting ducts^14^. The functional significance of caveolae for renal reabsorption of salt and water, however, remained to be elucidated further^11,14^. In this study we therefore utilized Cav1-deficient (Cav1−/−) mice to assess the contribution of caveolae to renal water and electrolyte handling. Epithelial as well as endothelial functions of Cav1 in the kidney have been addressed.

## Results

### Renal distribution of Cav1 and caveolae in WT and Cav1−/− mice

**Figure 1:**
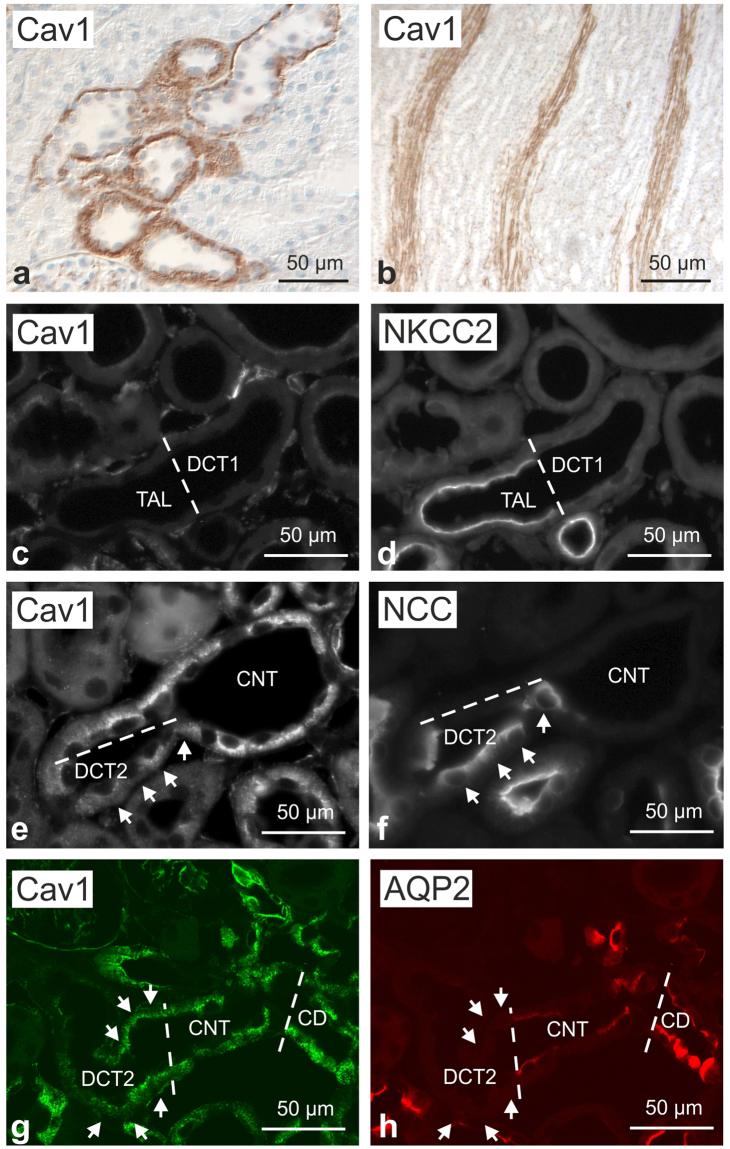
Renal distribution of caveolin 1 in wild-type mouse kidney. (**a**,**b**) Representative bright-field images showing basolateral caveolin 1 (Cav1) distribution along renal distal epithelia (**a**) as well as a Cav1 signal in medullary vasculature (**b**); immunoperoxidase/hematoxylin staining, interference contrast. (**c**,**d**) Double immunofluorescence staining for Cav1 and Na-K-2Cl cotransporter (NKCC2) shows the transition between NKCC2-positive thick ascending limb (TAL) and NKCC2-negative early distal convoluted tubule (DCT1; bar); TAL and DCT1 are Cav1-negative. (**e**,**f**) Immunofluorescence staining for Cav and, on a consecutive section, for DCT-specific Na-Cl cotransporter (NCC) showing the transition between NCC-positive late DCT (DCT2) and ensuing NCC-negative connecting tubule (CNT); moderate basolateral Cav1 signal is detected in DCT2 (arrows); stronger Cav1 signal is observed in CNT principal cells. (**g**,**h**) Double immunofluorescence staining for Cav1 and aquaporin 2 (AQP2) shows a moderate Cav1 signal in AQP2-negative DCT2 (arrows) and a stronger Cav1 signal in CNT and collecting duct (CD) principal cells (bars indicate DCT2/CNT and CNT/CD transitions).

**Figure 2:**
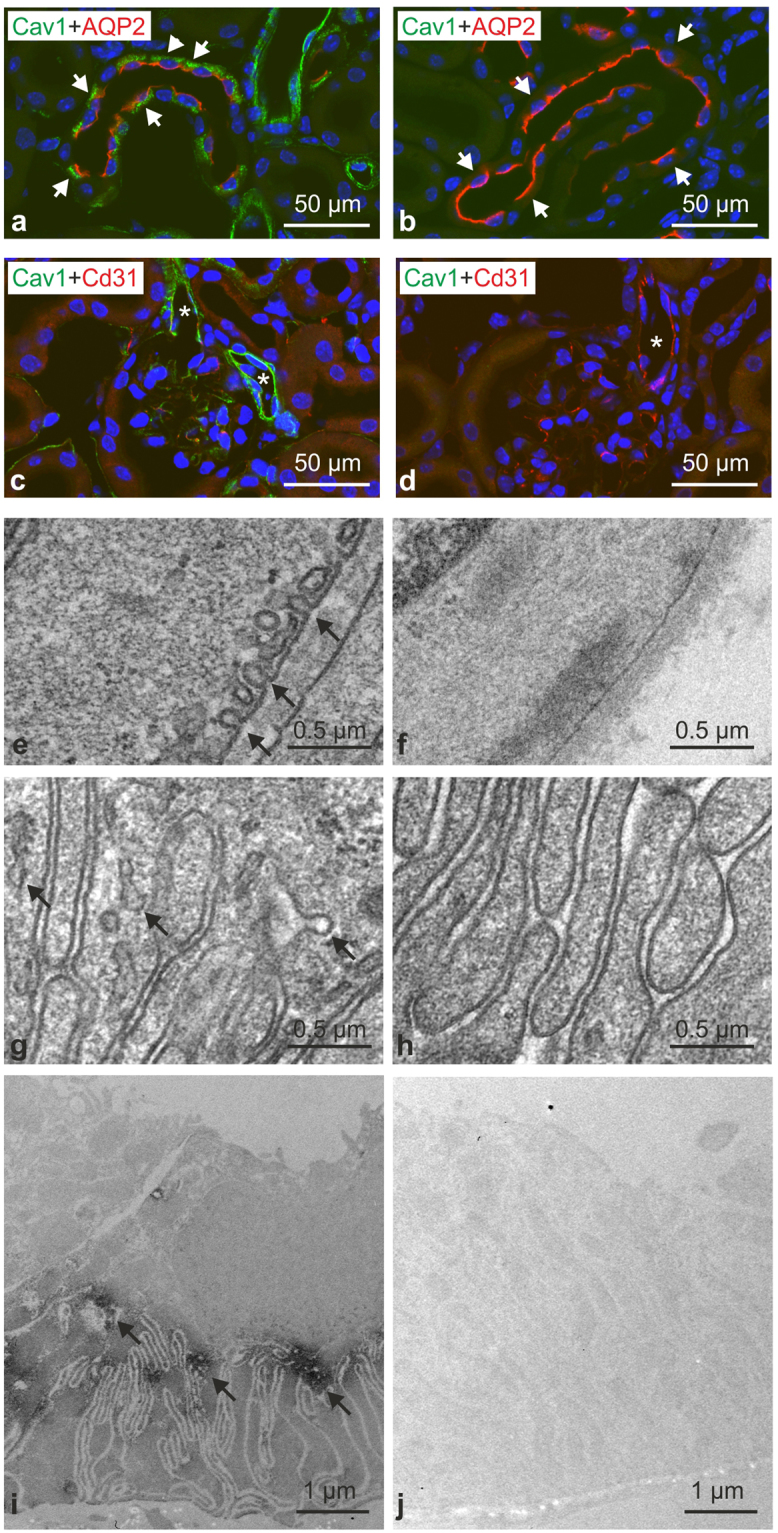
Verification of caveolin-1 deficiency. (**a**,**b**) Representative confocal images of control WT (a; n = 4) and Cav1-deficient kidneys (b; n = 4) after double immunofluorescence staining for Cav1 (green signal) and AQP2 (red signal) showing strong basolateral Cav1 labeling in WT CD principal cells identified by positive luminal AQP2 signals (exemplified by arrows); no corresponding Cav1 signal was detected in Cav1−/− kidneys (arrows). (**c**,**d**) Double staining for vascular Cav1 signal (green; arterioles marked with asterisks) and endothelial marker CD31 (red) in WT (**c**) and Cav1−/− kidneys (**d**); note strong Cav1 signal in WT but not in Cav1−/− vessels. (**e**–**h**) Representative transmission electron microscopy images of WT (**e**,**g**) and Cav1-deficient kidneys (**f**,**h**) documenting caveolae (arrows) in the plasma membrane of WT vascular smooth muscle (**e**) and CD principal cells (**g**), and absence of caveolae in Cav1-deficient vascular smooth muscle (**f**) and CD principal cells (**h**). (**i**,**j**) Pre-embedding labeling of WT and Cav1−/− kidney sections for Cav1 detected by transmission electron microscopy shows basolateral Cav1 labeling of principal CD cells in WT (arrows) but not in Cav1 kidneys.

In light of the scarce information available on Cav1 distribution in the mouse kidney, we first analyzed overall Cav1 expression in the renal parenchyma of WT mice. In an overview approach, anti-Cav1 immunoperoxidase staining showed a significant basolateral signal in a subpopulation of cortical distal tubules as well as in blood vessels such as the outer medullary vascular bundles (Fig. 1a,b). Double immunofluorescence staining for Cav1 and Na,K,2Cl-cotransporter (NKCC2) of the thick ascending limb (TAL) showed that the entire TAL and macula densa were negative for Cav1; beyond the macula densa, the transition between TAL and DCT showed that the initial distal convoluted tubule (DCT1) was Cav1-negative as well (Fig. 1c,d). On consecutive sections, co-staining of Cav1 and Na,Cl-cotransporter (NCC) demonstrated the onset of Cav1 expression in the late portion of the DCT (DCT2), and a stronger signal was also found in ensuing, NCC-negative connecting tubule (CNT) principal cells which were identified by morphological criteria (Fig. 1e,f). Double immunofluorescence staining for Cav1 and aquaporin 2 (AQP2) showed an additional, substantial Cav1 signal in the collecting duct (CD) principal cells (Fig. 1g,h). Cav1−/− kidneys showed no significant Cav1 signals in DCT2 or in CNT and CD principal cells (Fig. 2a,b). Renal blood vessels showed a Cav1 immunofluorescent signal in the arteries, arterioles, medullary vascular bundles, and capillaries of WT kidneys. There was pronounced staining of the arteriolar smooth muscle layer, and endothelia were positive throughout the vasculature, including glomerular capillaries, as revealed by double immunofluorescence staining with the endothelial marker CD31 (Fig. 2c). Cav1 staining was absent from the entire vasculature in Cav1−/− kidney (Fig. 2d). Ultrastructural analysis by transmission electron microscopy showed densely packed rows of caveolae along plasma membranes of vascular smooth muscle cells and endothelia in WT, but none in Cav1−/− kidneys (Fig. 2e,f). Caveolae were also found attached to the basolateral membrane of CNT and CD principal cells of WT, but not Cav1 −/− kidneys (Fig. 2g,h). In line with this, pre-embedding labeling of Cav1 and detection by transmission electron microscopy produced a signal along the basolateral membrane of principal CNT and CD cells in WT but not in Cav1−/− kidneys (Fig. 2i,j).

### Urine and blood analysis of Cav1−/− mice

For steady state analysis, mice were placed in metabolic cages to obtain 24 h urine samples. Plasma samples were obtained when mice were sacrificed for organ removal. Analysis of plasma electrolytes and creatinine levels revealed no significant differences between WT and Cav1−/− mice (Table 1). Urinary sodium excretion (+142%, p < 0.05), sodium/creatinine ratio (+94%, p < 0.05), fractional sodium excretion (+81%, p < 0.05), fractional chloride excretion (+107%, p < 0.05), as well as urine volume (+126%, p < 0.05) were significantly increased in Cav1−/− compared to WT mice (Table 1). There were no significant differences between WT and Cav1−/− mice with respect to potassium, calcium, urea, and creatinine levels; although a strong trend towards augmented calcium excretion and a moderate trend towards potassium wasting were observed. A parallel cohort of WT and Cav1−/− mice was subjected to water deprivation for 18 h to challenge their urinary concentrating ability. This experiment produced no statistical differences in urinary electrolyte excretion between the strains, showing only trends towards increased urinary volume and urinary levels of sodium, chloride, potassium and calcium in Cav1−/− mice (Table 2).

**Table 1:** Physiological steady state parameters, WT vs. Cav1−/− mice.

	WT (n = 6)	Cav-1−/− (n = 6)
*Plasma parameters (mean* ± *SD)*		
Na^+^, mmol/L	152.1 ± 1.5	143.7 ± 12.0
K^+^, mmol/L	5.9 ± 0.3	6.2 ± 0.8
Cl^−^, mmol/L		
Ca^2+^, mmol/L	1.88 ± 0.05	1.90 ± 0.03
Creatinine, mg/dl	0.11 ± 0.01	0.10 ± 0.01
Urea, mg/dl	54.5 ± 4.4	55.4 ± 7.4
*Urinary analysis* (*mean* ± *SD*)		
Body weight, g	28.3 ± 0.8	29.8 ± 0.8
Urinary volume, g/g body weight	0.023 ± 0.005	0.052 ± 0.010*
Na^+^ excretion, mmol/kg*24 h	2.6 ± 1.5	6.3 ± 1.5*
K^+^ excretion, mmol/kg*24 h	5.2 ± 1.6	8.2 ± 1.9
Cl^−^ excretion, mmol/kg*24 h	3.6 ± 0.9	7.7 ± 2.1
Ca^2+^ excretion, mmol/kg*24 h	0.07 ± 0.01	0.16 ± 0.06
Creatinine, mg/dl	41.2 ± 4.2	24.7 ± 5.1
Creatinine, mg/g body weight	0.009 ± 0.002	0.011 ± 0.001
Na^+^/creatinine ratio	33.7 ± 4.2	65.4 ± 12.1*
K^+^/creatinine ratio	66.4 ± 10.7	85.1 ± 17.2
Cl^−^/creatinine ratio	45.9 ± 4.6	80.6 ± 19.0
Ca^2+^/creatinine ratio	0.95 ± 0.09	1.64 ± 0.62
*Renal function (mean* ± *SD)*		
Fractional Na^+^ excretion, %	0.22 ± 0.03	0.40 ± 0.06*
Fractional K^+^ excretion, %	10.83 ± 1.68	12.68 ± 1.75
Fractional Cl^−^ excretion, %	0.42 ± 0.04	0.87 ± 0.13*
Fractional Ca^2+^ excretion, %	0.49 ± 0.05	0.86 ± 0.28
Na^+^/K^+^ ratio	0.57 ± 0.12	0.78 ± 0.06

*p < 0.05.

**Table 2:** Physiological parameters of WT vs. Cav1−/− mice upon water deprivation.

	WT (n = 5)	Cav-1−/− (n = 6)
*Urinary analysis* (*mean* ± *SD*)		
Body weight, g	28.5 ± 0.7	29.2 ± 0.7
Urinary volume, g/g body weight	0.028 ± 0.003	0.041 ± 0.008
Na^+^ excretion, mmol/kg *18 h	4.2 ± 1.1	5.9 ± 0.9
K^+^ excretion, mmol/kg *18 h	7.2 ± 1.2	8.1 ± 1.0
Cl^-^ excretion, mmol/kg *18 h	6.9 ± 1.1	8.0 ± 1.4
Ca^2+^ excretion, mmol/kg *18 h	0.07 ± 0.02	0.12 ± 0.03
Creatinine, mg/dl	38.2 ± 1.7	30.3 ± 3.5
Creatinine, mg/g body weight	0.011 ± 0.001	0.012 ± 0.002
Na^+^/creatinine ratio	45.1 ± 2.6	58.2 ± 6.4
K^+^/creatinine ratio	75.3 ± 6.6	81.2 ± 6.5
Cl^-^/creatinine ratio	72.7 ± 5.1	79.3 ± 12.7
Ca^2+^/creatinine ratio	0.72 ± 0.14	1.23 ± 0.24
Na^+^/K^+^ ratio	0.63 ± 0.07	0.73 ± 0.07

*p < 0.05.

### Epithelial effects of Cav1 deficiency

**Figure 3:**
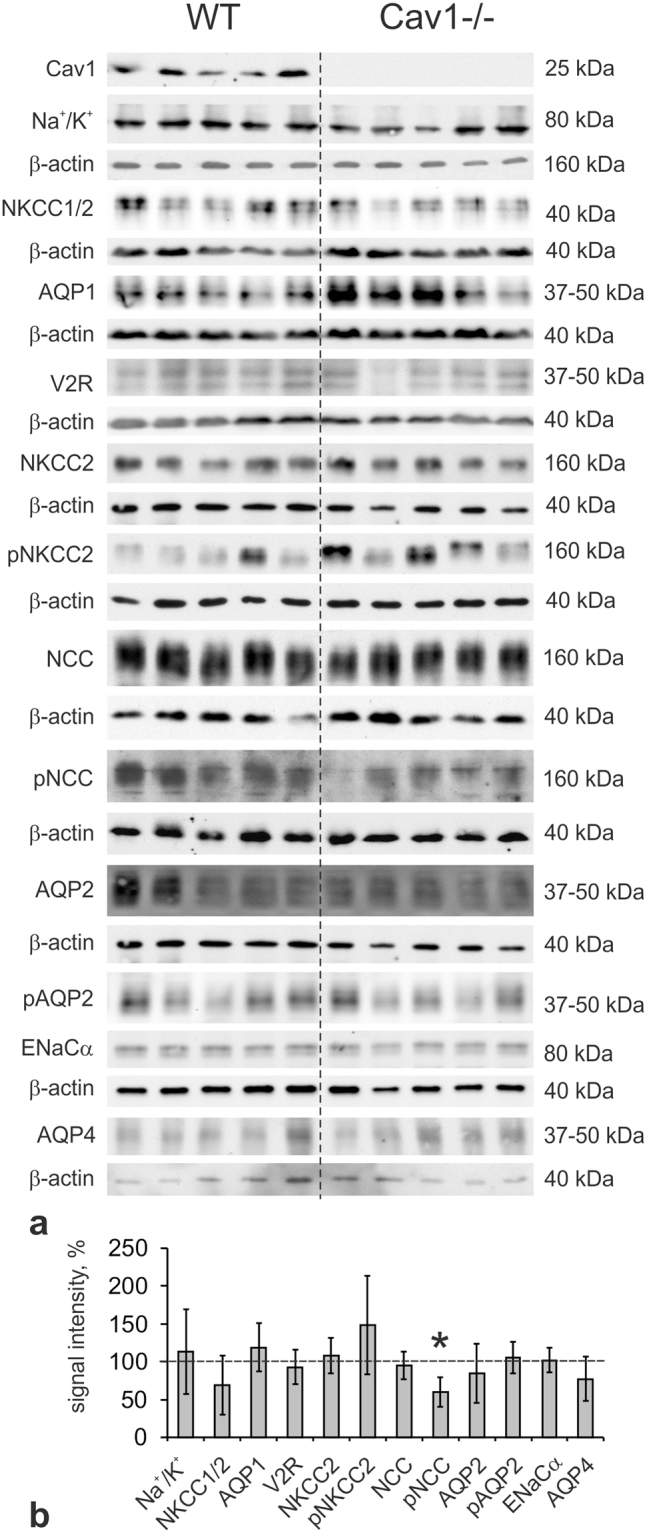
Effects of caveolin-1 deficiency on epithelial parameters by immunoblotting. (**a**) Representative immunoblots of WT (n = 6) and Cav1-deficient (Cav1−/−; n = 6) kidney lysates detected by antibodies to Cav1, alpha subunit of Na/K-ATPase (Na^+^/K^+^), NKCC1/2 (antibody recognizes both NKCC isoforms), AQP1, V2R, NKCC2, phosphorylated (p) NKCC2 (pT96/T101-NKCC2), NCC, pNCC (pS71-NCC), AQP2, pAQP2 (pS256-AQP2), alpha subunit of epithelial sodium channel (ENaCα) and aquaporin 4 (AQP4); β-actin serves as loading control below the respective blots; all molecular weight levels are approximate. (**b**) Densitometric quantification of the immunoreactive signals normalized to the respective loading controls. Data is expressed as the mean ± standard deviation; *p < 0.05 (Student’s t test for normal distribution); original immunoblot scans are available in Supplementary Data.

Next, we tested effects of Cav1-deficiency on the abundance of relevant distal transporters and channels by immunoblotting of whole kidney lysates. Protein levels of basolateral and luminal transporters and channels, including Na^+^/K^+^-ATPase, NKCC1, aquaporin 1 (AQP1), NKCC2, NCC, aquaporin 2 (AQP2), aquaporin 4 (AQP4), and the alpha subunit of the epithelial sodium channel (ENaCα), as well as of the basolateral vasopressin V2 receptor (V2R) did not differ between WT and Cav1−/− kidneys (Fig. 3a,b). Since the activities of AQP2, NKCC2 and NCC primarily depend on their phosphorylation at conserved threonine- or serine residues^15–17^, we further applied antibodies recognizing their phosphorylated species. AQP2 phosphorylation at S256 and NKCC2 phosphorylation at T96/T101 were not affected by Cav1-deficiency, whereas NCC phosphorylation at S71 was significantly decreased in Cav1−/− compared to WT mice (−40%, p < 0.05; Fig. 3a,b).

### Vascular effects of Cav1 deficiency

**Figure 4:**
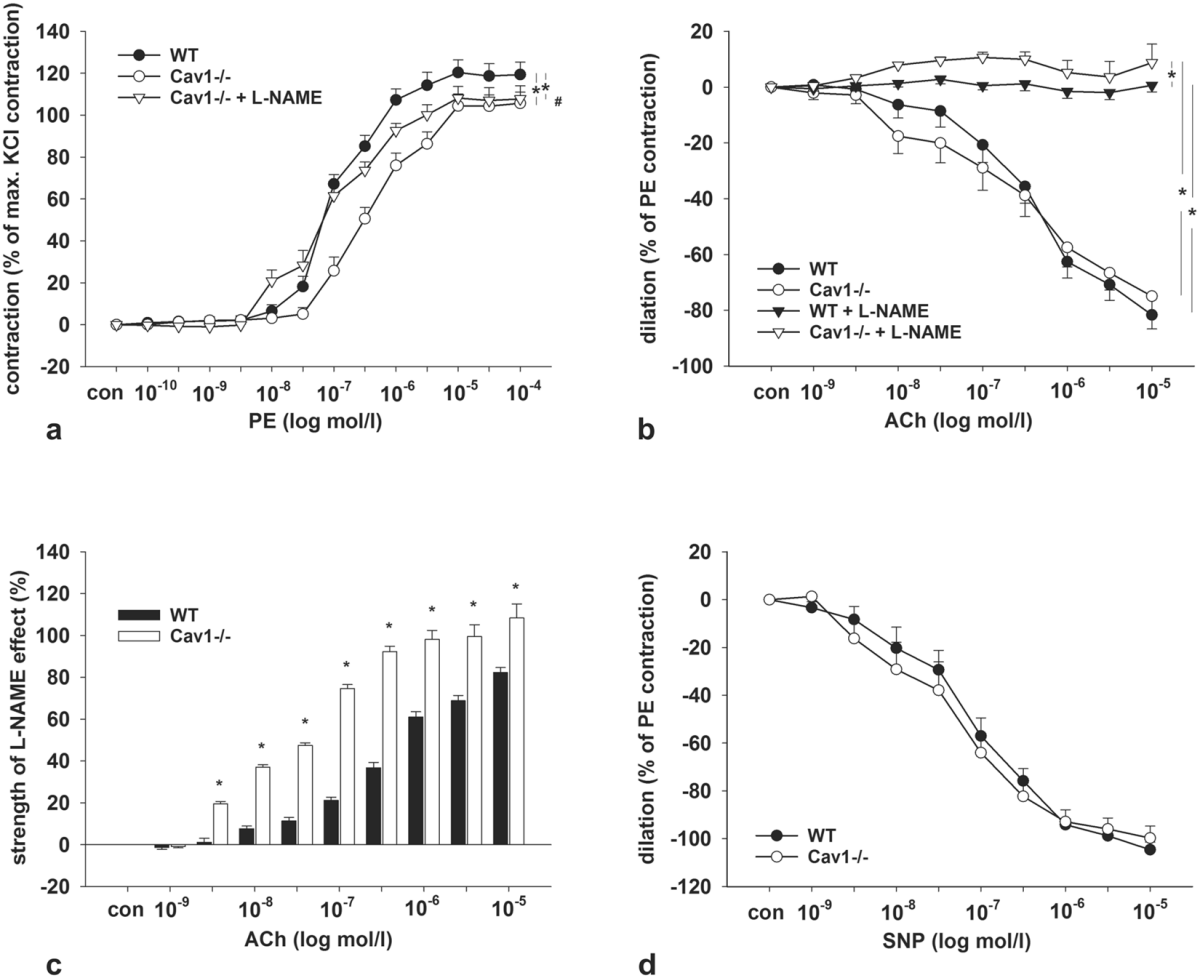
Effects of caveolin 1-deficiency on arterial contraction and relaxation. (**a**) Phenylephrine (PE) cumulative concentration response curves (10^–10^ to 10^−4^ mol/l) in WT (n = 13) and Cav1-deficient mice (Cav1−/−; n = 12) with and without L-NAME pretreatment (n = 10 and n = 13, respectively). (**b**) Acetylcholine (ACh, 10^−9^ to 10^−5^ mol/l) cumulative concentration response curves in WT (n = 16) and Cav1−/− (n = 14) with and without L-NAME pretreatment (n = 10 and n = 9, respectively). (**c**) Effects of L-NAME pretreatment on the vascular tone during ACh application (calculated from data in Fig. 4b). (**d**) Sodium nitroprusside (SNP, 10^−9^ to10^−40^ mol/l) cumulative concentration response curves for WT (n = 18) and Cav1−/− (n = 15). Data are expressed as the mean values ± standard deviations, *p < 0.05. *Indicates significant differences between groups (ANOVA like Brunner Test for non-normal distribution), ^#^ Indicates significant differences between Cav1−/− and Cav1−/− + L-NAME. (ANOVA, Student’s test for normal distribution and post hoc Mann Whitney test for independent groups).

**Figure 5:**
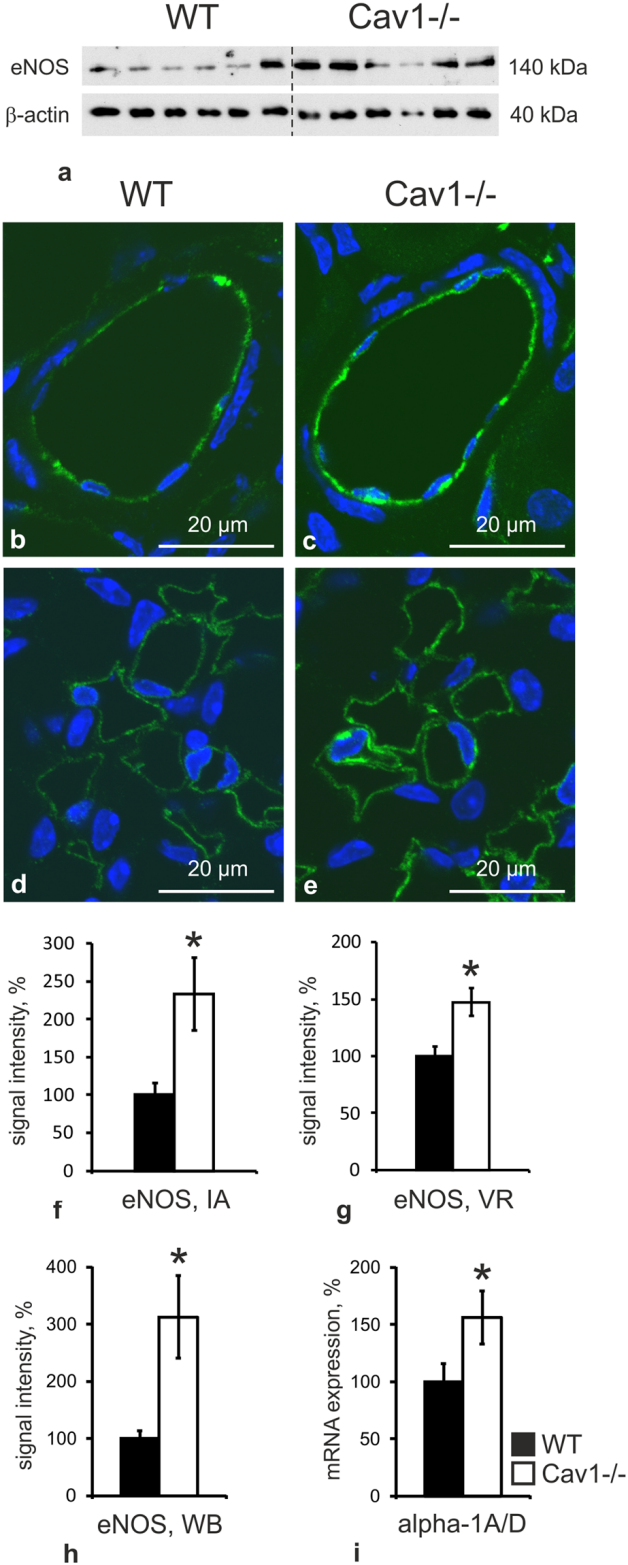
Effects of caveolin 1-deficiency on eNOS and alpha-1 adrenergic receptors. (**a**) Representative immunoblot of WT (n = 6) and Cav1-deficient (Cav1−/−; n = 6) kidney lysates detected by antibodies to endothelial nitric oxide synthase (eNOS) and β-actin as loading control; indicated molecular weights are approximate. (**b**,**c**) Representative confocal images of interlobar arteries showing increased endothelial eNOS signal (green) in Cav1−/− (n = 4) compared to WT kidneys (n = 4). (**d**,**e**) Vasa recta also show an enhanced eNOS signal in Cav1-/- kidney. Nuclei are counter-stained with DAPI (blue). (**f**,**g**) Quantification of confocal eNOS signals in interlobar arteries (IA) and vasa recta (VR) by intensity. (**h**) Densitometric quantification of eNOS immunoblots shown in A, normalized to loading controls. (**i**) Evaluation of alpha-1 adrenergic receptor mRNA expression in WT vs. Cav1−/− kidneys using quantitative PCR. Data is expressed as the mean values ± standard deviations, *p < 0.05 (Student’s t test for normal distribution); WB – Western blot, IF – immunofluorescence.

Since caveolae have been implicated in the regulation of vascular tone and reactivity, we evaluated contractile and dilatory responses in renal interlobular arteries obtained from WT and Cav1−/− kidneys. Cumulative addition of phenylephrine (PE) to the bath solution revealed significantly reduced sensitivity (EC_50_: 422 ± 65 nM in WT vs. 105 ± 9 nM in Cav1−/−, p < 0.05) and reduced maximum contractile responses in Cav1−/− arteries compared to WT arteries (119 ± 6% in WT vs. 106 ± 5% in Cav1−/−, p < 0.05; Fig. 4a). Pre-incubation of vessels with L-NAME to inhibit the production of nitric oxide (NO) improved the contractile response to PE in Cav1−/− arteries, suggesting increased NO bioavailability upon Cav1 disruption (EC_50_: 112 ± 41 nM, p < 0.05; Fig. 4a). Further, we tested endothelium-dependent and independent relaxation responses using acetylcholine (ACh) and sodium nitroprusside (SNP), respectively. ACh applied after preconstriction with PE produced similar relaxation responses in both genotypes, which could be abolished by preincubation of the arteries with L-NAME (p < 0.05), but the effect of L-NAME was more pronounced in Cav1−/− compared to WT arteries (p < 0.05), again suggesting increased NO levels in Cav1−/− arteries (Fig. 4b,c). The endothelium-independent relaxation tested by cumulative application of SNP was not different in WT and Cav1−/− arteries (Fig. 4d). In line with the increased response of Cav1−/− arteries to the NOS-inhibitor L-NAME, immunoblotting of kidney lysates showed enhanced abundance of the endothelial nitric oxide synthase isoform (eNOS) in Cav1−/− compared to WT kidneys (+213%, p < 0.05; Fig. 5a,h). This result was corroborated by confocal microscopic evaluation of WT and Cav1−/− kidneys showing enhanced eNOS immunoreactive signal in vascular endothelia including interlobar arteries and vasa recta (+133% in interlobar arteries and +47% in vasa recta; Fig. 5b–g). We further evaluated the renal expression of alpha-adrenergic receptors by quantitative PCR and found a significant increase in Cav1−/− compared to WT kidneys (+63%, p < 0.05; Fig. 5i) which may reflect a compensatory response to enhanced NO bioavailability. Together, these results point to an attenuated vasoconstriction along with increased endothelial NO bioavailability under Cav1−/− deficiency.

### Effects of CGL4-causing PTRF mutation in cell culture

**Figure 6:**
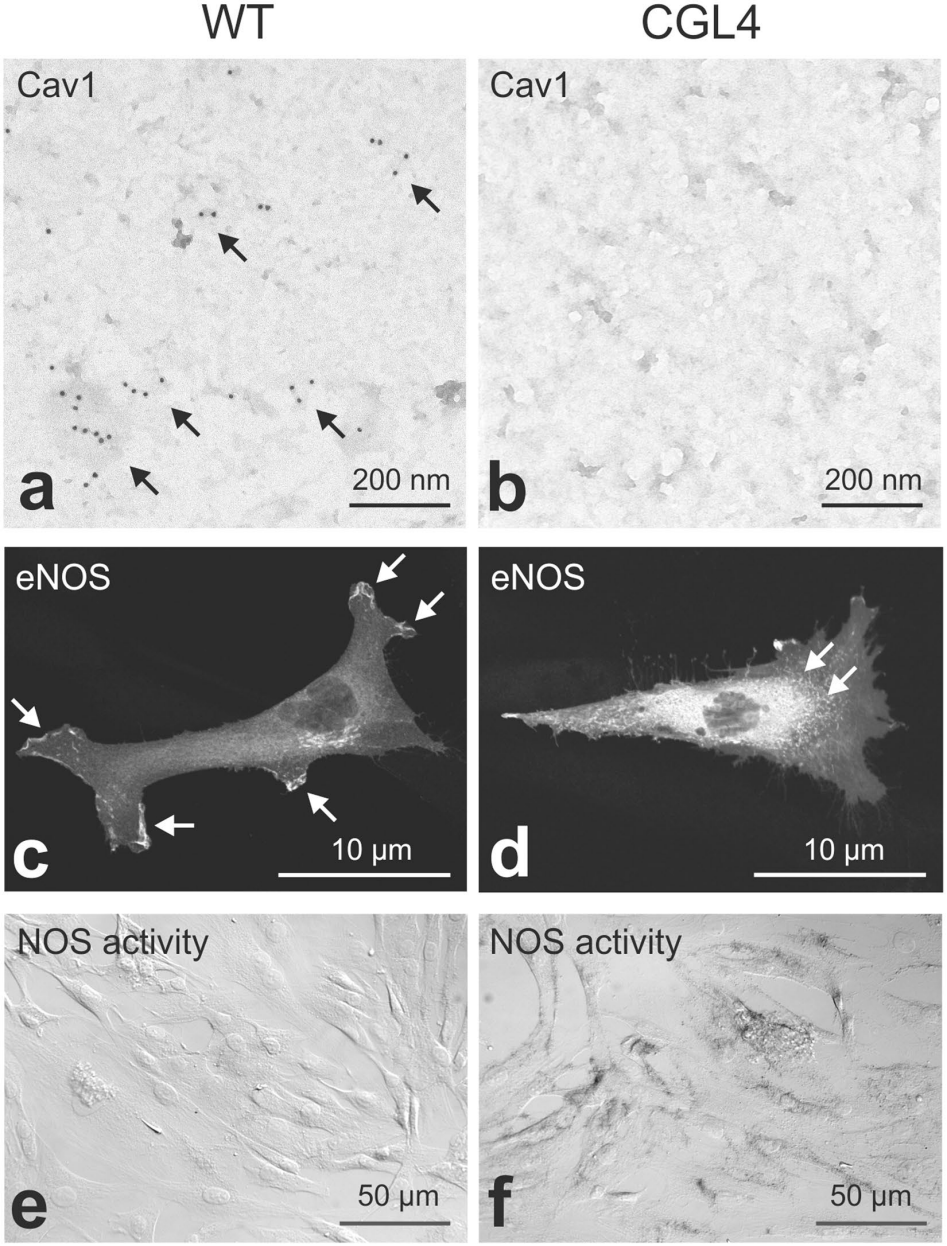
Cellular distribution of eNOS and NADPH diaphorase activity in WT vs. CGL4 fibroblasts. (**a**,**b**) Immunogold detection of Cav1 (**a**; 10 nm gold grains, arrows) in plasma membrane sheets obtained by rip/flip-technique. Note that in CGL4-fibroblasts, membrane is virtually devoid of a Cav1 signal (B); scale bars 100 nm. (**c**,**d**) Cellular distribution of transfected eNOS with a membrane-bound signal in WT (c, arrows) as opposed to the more perinuclear signal in CGL4-fibroblasts (d, arrows); scale bars 10 µm. (**e**,**f**) Enhanced NADPH-diaphorase activity in CGL4-fibroblasts (**f**) suggests enhanced NOS activity in Cav1 deficiency; representative results of three independent experiments.

Next, we utilized fibroblasts from patients with a CGL4-causing mutation of PTRF (CGL4-fibroblasts) to study effects of caveolar disruption on the cellular distribution of eNOS. To this end, CGL4- and wild type (WT) fibroblasts were transfected with eNOS. Ultrastructural analysis of plasma membrane fragments obtained by the rip/flip method^18^ and labeled for Cav1 revealed abundant Cav1-positive domains in the plasma membrane of WT but not of CGL4-fibroblasts (Fig. 6a,b). This result therefore confirms that the CGL4-causing mutation of PTRF is associated with impaired formation of caveolae, as reported previously^7^. Transfecting the cells with GFP-tagged eNOS resulted in a substantial association of eNOS with plasma membrane in WT cells, whereas CGL4-cells showed predominantly intracellular accumulation of eNOS (Fig. 6c,d). Evaluation of NOS activity using the histochemical NADPH diaphorase reaction produced stronger signal in CGL4-fibroblasts as compared to control cells (Fig. 6e,f). This data suggests that depletion of caveolae enhances the cytoplasmic eNOS fraction, which probably facilitates NO biosynthesis^19^.

## Discussion

The present results expand upon previous work on the renal distribution of Cav1 and caveolae and suggest that Cav1-deletion may affect renal performance at multiple levels. High-resolution immunohistochemistry revealed abundant Cav1 expression in the renal epithelia and vasculature. The epithelial Cav1 signal was restricted to the distal nephron epithelia comprising the late DCT and CNT/CD principal cells, which mediate reabsorption of divalent cations and participate in renal potassium handling^20,21^. Previous evaluation of an alternative Cav1-knockout mouse line reported significant urinary calcium and potassium losses in Cav1-deficient mice, whereas in the present study only a trend towards enhanced urinary calcium and potassium excretion was observed upon Cav1 disruption^12,13^. We believe that the lack of statistical significance for calcium and potassium values is due to relatively small sample sizes in our animal experiments (6 animals per group). The milder phenotype of Cav1-deletion in our study may also be related to inherent differences between the distinct knockout-strains^5,12,13^. Steady state polyuria and urinary salt loss in our Cav1−/− mice suggest that Cav1 may further be involved in the renal reabsorption of salt and water. Analysis Na^+^/K^+^-ATPase, which can be functionally modulated by Cav1^22^, revealed its unchanged renal abundance and further relevant salt and water transport proteins of the proximal and distal nephron showed no major quantitative alterations upon Cav1-disruption, except for a moderate reduction of phosphorylated NCC. Since the analyzed phosphorylation site (S71) is essential for NCC activation, this result may, at least in part, account for the urinary sodium loss^15^. Mechanistic molecular links between basolateral Cav1 and apical NCC are elusive, especially in view of their co-expression only in the relatively short late DCT portion. However, due to association of Cav1 with calcium reabsorption in the distal nephron, its deficiency may trigger local or systemic compensatory mechanisms suppressing NCC in favor of more efficient calcium reabsorption, as observed with pharmacologic inhibition of the transporter by thiazides or during action of the parathyroid hormone^23,24^. Apart from NCC, functional effects of Cav1-deficiency on transporters and channels of principal CNT/CD cells deserve more precise characterization in future studies. The present analyses did not reveal changes in ENaCα abundance upon Cav1 disruption and the urinary Na^+^/K^+^ ratio was not significantly changed, which suggested preserved ENaC function. However, in view of reported functional changes of basolateral potassium transport along the distal nephron of Cav1−/− mice^13^, the Na^+^/K^+^ ratio alone is insufficient for robust assessment of ENaC function. Therefore, functional evaluation of ENaC activity in the future would be helpful to clarify this issue. Interestingly, water deprivation for 18 h abolished differences in urinary electrolyte excretion between WT and Cav1−/− mice suggesting that Cav1-deficiency can be efficiently compensated upon challenge. Water deprivation elicits increases of endogenous vasopressin (AVP) levels thereby promoting salt and water reabsorption via activation of V2R along the distal nephron and in principal CD cells^17,25,26^. Since V2R expression was not altered in Cav1−/− mice, increased AVP levels upon water deprivation with resulting V2R-dependent stimulation of distal transporters and channels may contribute to compensation of Cav1-deficiency along with V1a receptor-induced vasoconstriction^27^. Moreover, AVP has been shown to interfere with both epithelial and vascular NO systems^27–29^.

Vascular effects of Cav1-deficiency were assessed in isolated renal arteries. Cav1-disruption was associated with reduction of their contractile response to the α_1_-agonist PE, unchanged relaxation after ACh application, but stronger effect of L-NAME on vascular tone during ACh application. When assuming an increased NO bioavailability in Cav1−/− animals, a stronger effect of ACh, which seems to act predominantly via NO release in these arteries, should be expected. However, WT and Cav1−/− vessel presented similar and powerful responses to cumulatively increasing concentrations of ACh. This data is in contrast to the markedly stronger relaxation to ACh-bolus application reported in Cav1-deficient arteries of the same knockout strain^5^. This discrepancy may be related to different types of protocols (bolus vs. cumulative application) as well as the varying types of the arteries being studied in the present vs. previous work. The reduced sensitivity to PE supports the idea of an activated NO system in Cav1−/− mice, although preserved or even enhanced contractile response to α_1_-receptor agonists have been previously reported in mesenteric arteries and aorta upon Cav1 or PTRF disruption, respectively^5,30^. Physical and functional association of caveolae with adrenergic receptor subtypes was described in cardiac myocytes^31–33^. However, disruption of caveolae in isolated rat tail arteries using cholesterol depletion did not affect their contractile response to adrenergic stimulation^34^. Therefore, the role of caveolae in mediating adrenergic stimulation remains to be clarified. Our present data showing reduced PE-induced contractility in Cav1-deficient renal arteries may reflect increased NO bioavailability with resulting attenuation of vasoconstriction, rather than direct inhibition of the adrenergic system by caveolae disruption. In this light, increased expression of α_1_-adrenergic receptors in Cav1−/− kidneys observed in the present study may reflect a compensatory reaction serving to balance enhanced NO bioavailability, although their abundance at the protein level in renal vessels still needs to be studied. Compensatory mechanisms related with increased NO bioavailability would also help to explain the moderately higher contractile tone of Cav1−/− arteries upon pretreatment with L-NAME in experiments testing endothelium-dependent relaxation using ACh.

Inhibitory effects of caveolae or Cav1 on the activity of NOS isoforms have been reported in a number of previous studies^35–39^. With respect to the kidney, an association between Cav1 and eNOS has been proposed to play a role in the pathogenesis of diabetic nephropathy^40,41^. Nitric oxide derived from eNOS has further been shown to promote diuresis through vascular and epithelial effects in the kidney^29^. Cav1 disruption may therefore increase NO bioavailability, which in turn may contribute to the observed polyuria in the Cav1−/− mice. The increased abundance of eNOS in Cav1−/− kidneys and reduced contractility of Cav1−/− interlobular arteries observed in this study provide indirect evidence for enhanced NO release upon Cav1 disruption. This would also agree with the reported increase of NO release in Cav1-deficient aorta^5^. The underlying mechanisms may include direct inhibition of eNOS activity by the protein network of caveolae as well as enhanced internalization and degradation of eNOS via interactions with its trafficking factor NOSTRIN and Cav1 directing the enzyme to caveosomes^36,42^. Regulation of eNOS activity appears to be closely linked to its cellular distribution^42,43^. Activating Golgi-associated eNOS requires protein kinase B, whereas plasma membrane-associated eNOS responds to changes in calcium-dependent signaling^43,44^. Cytosolic localization of eNOS has been associated with its activation^45,46^. To extend information on caveolae-dependent eNOS regulation we have studied the cellular distribution of transfected eNOS in human fibroblasts carrying CGL4-causing PTRF mutation^7^. The resulting depletion of caveolae was associated with perinuclear accumulation and reduced targeting of eNOS to the plasma membrane which, we assumed, would indicate changes in its activity^43,45^. Indeed, indirect evaluation of NOS activity using histochemical NADPH diaphorase staining demonstrated enhanced endogenous NOS activity in the caveolae-deficient CGL4-fibroblasts. This data further corroborates the role of caveolae in the regulation of eNOS activity and is in line with other results of our study, documenting increased eNOS function in Cav1-deficient kidneys.

Increased vascular NO production may have paracrine effects on adjacent transporting epithelia, primarily in the medulla^47,48^. Increased bioavailability of NO has been reported to attenuate salt reabsorption along the distal nephron chiefly due to inhibition of NKCC2 activity^29,49^. However, NKCC2 abundance and phosphorylation were not reduced in Cav1−/− kidneys. Therefore, changes in local NO production in Cav1−/− renal vessels were likely not strong enough to induce significant paracrine effects on renal epithelia.

Caveolae have also been implicated in the regulation of detrusor contractility, which may have effects on urine flow^50,51^. However, manifestation of impaired detrusor function was evident only in old mice lacking Cav1 (1-year-old), whereas young mice (up to 3-month-old) did not show significant changes^51^. Therefore, alterations of urinary bladder function in the mice used in the present study are unlikely.

In summary, our study demonstrates that renal caveolae, which depend on Cav1 expression, are involved in the control of salt and water reabsorption. Absence of renal caveolae is associated with moderate salt loss and enhanced urine flow. In the tubular compartment, a decrease in activating NCC phosphorylation upon Cav1-deletion may explain diminished electrolyte reabsorption. In the vascular compartment, lack of caveolae is associated with disinhibition of eNOS, resulting in increased NO bioavailability and decreased vascular contractility, which aligns with impaired volume conservation. Since caveolins and caveolae have been recognized as potential targets for pharmaceutical interventions^52^, our data may have clinical implications.

## Methods

All methods were performed in accordance with the relevant guidelines and regulations, such as standards of Good Scientific Practice and permissions of local authorities where applicable.

### Animal experiments

Generation of Cav1-deficient mice has been described previously^5^. All animal experiments were approved by the Regional Office for Health and Social Affairs Berlin (LAGESO permission: G0220/12). For physiological evaluation of baseline kidney performance 10–14 weeks old male wild type (WT; n = 6) and Cav1−/− mice (n = 6) were kept in metabolic cages for 24 h at chow and water ad libitum to collect urine samples. After the metabolic cages blood and kidneys were collected under ketamine/xylazine-anaesthesia and mice were sacrificed by cervical dislocation. A parallel cohort of mice (5 WT and 6 Cav1−/− mice) was subjected to water deprivation for 18 h at chow ad libitum and urine samples were collected in metabolic cages. Plasma and urinary electrolytes were measured by routine automatic photometric methods (Cobas 8000, Roche Diagnostics) and fractional excretion of electrolytes was calculated [for example FE_Na_ = 100 × (Na_urinary_ × Crea_plasma_)/(Na_plasma_ × Crea_urinary_)]; kidneys were removed and processed for biochemical analysis. For morphological evaluation WT (n = 4) and Cav1−/− mice (n = 4) were anaesthetized by intraperitoneal injection of pentobarbital sodium (100 mg/kg body weight) and kidneys were fixed by retrograde perfusion with 3% paraformaldehyde/PBS via the abdominal aorta, removed, and processed for cryo-sectioning, paraffin-embedding, and LR White-embedding.

### Evaluation of vascular contraction and relaxation

16–20 weeks old male WT (n = 18) and Cav-1−/− mice (n = 16) were sacrificed by cervical dislocation after short anaesthesia using isoflurane, kidneys were removed and placed in ice-cold Krebs-Henseleit physiological solution (KHS; 118.6 mM NaCl, 4.7 mM KCl, 2.5 mM CaCl2, 1.2 mM MgSO4, 1.2 mM KH2PO4, 25.1 mM NaHCO3, 11.1 mM glucose and 0.02 mM EDTA)^53^. Up to four renal interlobar arteries were obtained per animal for different treatment protocols and mounted on a 40 µm stainless steel wire in a small vessel myograph (model 500 A; DMT, Aarhus Denmark) at 95% O2/5% CO2 gas mixture and 37 °C. Arterial force was recorded using a Powerlab 4/25 T data-aquisition system (ADInstruments, Castle Hill, New South Wales, Australia). The resting tension was set according to Mulvany’s normalization procedure^54^. The diameter was set to 80% of that calculated for a transmural tension of 100 mmHg. The procedure was performed without using relaxing substances. Maximal contraction of arterial vessels was induced by 100 mM KCl prior to each experiment and the obtained values were used as a standard for comparative evaluation of other vasoconstrictors. Concentration response curves (CRCs) for renal interlobar arteries were obtained by adding of cumulative doses of phenylephrine (10^−10^–3.10^−7^ M) to the bath solution. To obtain relaxation responses, vessels were pre-contracted to 50% of the maximum, KCl-induced contraction using phenylephrine at an appropriate concentration. The endothelium-dependent relaxation was evaluated by means of CRC to acetylcholine (ACh: 10^−9^–10^−6^ M) applied on top of phenylephrine. To assess the role of NO in the vascular tone, arteries were pretreated with *N*^G^-nitro-l-arginine methyl ester (l-NAME) 10^−4^ M for 15 min before measuring the ACh concentration response curves during preconstriction with phenylephrine. The endothelium-independent relaxation was investigated by CRC to sodium nitroprusside (SNP; 10^−9^–10^−4^ M) on top of phenylephrine.

### Cell culture

CGL4-fibroblasts were derived from a patient with CGL4 and control wild type fibroblasts were obtained from diagnostic samples for numeric chromosomal aberrations that showed no pathology^7^. For immunoblotting, fibroblasts were grown to confluence in DMEM in the presence of 15% FCS and penicillin/streptomycin on petri-dishes, washed with PBS, and harvested by mechanical scrapping in homogenization buffer (250 mM sucrose, 10 mM triethylamine and protease inhibitor [Complete; Roche, Mannheim, Germany]). Overexpression of eNOS in CGL4- and WT fibroblasts was induced by transient transfection of pcDNA3 plasmid containing GFP-tagged eNOS (Plasmid #22444; Addgene, Cambridge, MA) using JetPEI transfection reagent (Polyplus, Illkirch, France). After transfection, cells were incubated for 48 hours at 37 °C. For immunofluorescence, cells were grown on uncoated cover slips, washed with PBS, fixed in 4% PFA, and evaluated by confocal microscopy.

### Antibodies

The following primary antibodies were applied for immunofluorescence, immunohistochemistry, or immunoblotting: anti-AQP1 (Alpha diagnostic international, San Antonio, Texas, USA), anti-AQP2 (Santa Cruz Biotechnology, Heidelberg, Germany), anti-phospho-aquaporin 2 (pS256)^55^, anti-β-actin (Sigma-Aldrich, St. Louis, USA), anti-Cav1 (Santa Cruz Biotechnology, Heidelberg, Germany), anti-NKCC1 (T4; Developmental Studies Hybridoma Bank, University of Iowa, USA), anti-vasopressin V2 receptor^56^, anti-eNOS (Santa Cruz Biotechnology), anti-Na^+^/K^+^-ATPase (Millipore, Darmstadt, Germany), anti-NCC, anti-NKCC2, anti-phospho-NKCC2 (pT96/pT101), and anti-phospho-NCC (pS71)^57^.

### Immunofluorescence and immunohistochemistry

Paraffin-embedded kidney sections were dewaxed and boiled in citrate puffer (pH = 6) for 6 min to perform antigen retrieval. Cryo-sections and coverslips with fixed cultured cells were incubated in 0.5% Triton X-100 during 30 min for antigen retrieval. After a washing in PBS, kidney sections or cultured cells were incubated with 5% skim milk in PBS to block unspecific protein interactions and respective primary antibodies were applied for 1 h at room temperature followed by overnight incubation at +4 °C. By double-labelling the primary antibodies were applied consecutively, separated by a washing step. Signals were generated using fluorescent Cy2- or Cy3-conjugated (Dianova, Hamburg, Germany) or HRP-conjugated secondary antibodies (Sigma-Aldrich, St. Louis, USA) and evaluated using an LSM 5 Exciter confocal microscope (Carl Zeiss Microscopy GmbH) equipped with 40×/63 × EC Plan-NEOFLUAR oil-immersion objectives (N.A. 1.3/1.4). Filters for Excitation/Emission were set to 488/BP 505-550 for Cy2 and 543/BP 560–615 for Cy3 (BP = bandpass). Evaluation of confocal eNOS signal intensities in renal vessels conducted in kidney sections of WT and Cav1−/− mice (n = 3 in each group, at least 10 vascular profiles per animal) using ImageJ software. Background values obtained over the nuclei served as threshold and were subtracted from the respective signal levels.

### Immunoelectron microscopy of plasma membrane sheets

Plasma membrane sheets for electron microscopic analysis were prepared. Briefly, CGL4- and WT fibroblasts were grown to confluence on glass coverslips, fixed for 15 min in 0.5% paraformaldehyde/PBS, washed in PBS, and subsequently inverted on glow-discharged nickel electron microscopy grids coated with poly-L-lysine. Adherence of plasma membranes to the grid surface was forced by applying a gentle pressure to the coverslip for 15 s using a fine pair of forceps. The coverslips were then lifted leaving portions of the upper cell surface adherent to the poly-l-lysine-coated grid obtained as previously described^18,58^. The grids with adherent membrane fragments were then transferred to buffered 2% paraformaldehyde fixative solution for 20 min at room temperature and labeled with anti-Cav1 primary antibody and 10-nm gold-conjugated secondary antibody (Abcam). Grids were then fixed in 2% glutaraldehyde in PBS, contrasted with 1% aqueous tannic acid and 1% aqueous uranyl acetate, washed with distilled H_2_O, and examined by transmission electron microscopy (Zeiss E905).

### Ultrastructural analysis

For ultrastructural analysis of renal morphology perfusion-fixed WT and Cav1−/− kidney were subjected to additional fixation in 0,5% glutaraldehyde/PBS overnight at + 4 °C, processed for embedding using Epoxy Embedding Medium kit (Sigma-Aldrich, St. Louis, USA), and analyzed by transmission electron microscopy (Zeiss E905 or Technai^TM^ G2). Cellular distribution of Cav1 was analyzed by the pre-embedding technique. To this end, 30 µm thick cryostat sections from WT and Cav1−/− mice were treated with 0.5% Triton X-100 for 30 min, blocked with 5% skim milk in PBS for 30 min, and incubated with anti-Cav1 antibody for 1 h at room temperature followed by overnight incubation at + 4 °C. The corresponding HRP-conjugated secondary antibody was used for signal generation and the sections were processed for embedding in LR White resin, cut, and analyzed by transmission electron microscopy.

### Immunoblotting

Kidneys and cultured cells were homogenized mechanically in buffer containing 250 mM sucrose, 10 mM triethylamine and protease inhibitor (Complete, Roche, Mannheim, Germany) followed by short sonication on ice. Nuclei were removed by centrifugation at 1000xg for 10 minutes at 4 °C and the supernatants separated by polyacrylamide gel electrophoresis (50 µg protein/lane as determined by a BCA protein assay reagent kit [Pierce]; 8 to 10% gel). After electrophoretic transfer to polyvinylidene fluoride membranes, blocking was performed using 5% BSA/PBS or 5% milk/PBS and membranes were incubated with respective primary antibodies for 1 h at room temperature, followed by overnight incubation at 4 °C and subsequent exposure to HRP-conjugated secondary antibodies for 2 h at room temperature. Immunoreactive bands were detected by chemiluminescence, exposed to X-ray films, and the signals evaluated densitometrically. All data was normalized for expression of the housekeeping gene β-actin detected by monoclonal mouse anti-β-actin antibody (Sigma-Aldrich, St. Louis, USA).

### NADPH-diaphorase activity assay

For histochemical demonstration of nitric oxide synthase (NOS) tissue activity, the NADPH-diaphorase reaction was performed as described^59^. Briefly, 5 µm cryostate sections were incubated in 0.1 phosphate buffer containing nitro blue tetrazolium (NBT), b-NADPH, and Triton X-100. The optimal exposure time was set at 25 min at 37 °C. The reaction was stopped by rinsing the coverslips in PBS and the coverslips evaluated using a Leica DMRB microscope equipped with a SPOT 32 camera and MetaView 3.6a software (Diagnostic Instruments; Universal Imaging).

### mRNA Extraction, cDNA Synthesis and Quantitative PCR analysis

RNA from tissue or cell lysates was extracted using TRIzol reagent (Invitrogen, Darmstadt, Germany) according to the manufacturer’s protocol. cDNA was synthesized by reverse transcription (BioScript, Bioline, Luckenwalde, Germany) and quantitative PCR was performed using HOT FIREPol EvaGreen qPCR Mix Plus (Solis BioDyne, Tartu, Estonia) and specific primers for alpha 1 A/D receptor (forward primer: 5′-CTG CCA TTC CTC GTG AT-3′; reverse primer: 5′-GGC TGG AGC ATG GGT ATA TG-3′) or GAPDH in the Real Time PCR System 7500 (Applied Biosystems, Darmstadt, Germany). All samples were analysed in triplicate. Quantification and normalization of the threshold cycle (*C*_*t*_) was performed against GAPDH (Δ*C*_*t*_) with subtraction of the calibrator (ΔΔ*C*_*t*_) and the relative quantification (comparative *C*_*t*_ method) was performed by exponentiation, calculated using 2 to the power of ${{}^{{\rm{\Delta }}{\rm{\Delta }}}C}_{t}$ as described^60^.

### Analysis of data

All results are expressed as the mean ± SD. Data was analysed for normal distribution using the Shapiro-Wilk test and Q-Q plot (RStudio, version 1.0.143) or ANOVA. Unpaired 2-tailed *t* tests were used to compare two groups for immunohistochemical and immunoblot analysis. Statistical analysis of EC_50_ was performed by using an unpaired Mann-Whitney-Test and the courses of the concentration response curves were tested by applying an ANOVA like test for repeated measurements in non-normal distributed data (Brunner-test, The program is available: The R-project, http://www.r-project.org.). A *P*-value less than 0.05 was considered significant.

## Electronic supplementary material

Supplementary Information
