# Supplementary material for: Caveolin 1 Promotes Renal Water and Salt Reabsorption
Source: Sci Rep. 2018 Jan 11;8:545. doi: 10.1038/s41598-017-19071-6 (PMC5765123; doi:10.1038/s41598-017-19071-6)
Supplement: Supplementary file 1 — Supplementary Information [file 41598_2017_19071_MOESM1_ESM.pdf]

Caveolin 1 Promotes Renal Water and Salt Reabsorption

Yan Willière[1,§], Aljona Borschewski[1,§], Andreas Patzak[2], Tatiana Nikitina[2], Carsten Dittmayer[1], Anna L. Daigeler[1], Markus Schuelke[3], Sebastian Bachmann[1], and Kerim Mutig[1,*]

[1]Department of Anatomy, Charité-Universitätsmedizin Berlin, Berlin, Germany;
[2]Department of Physiology, Charité-Universitätsmedizin Berlin, Berlin, Germany
[3]Department of Neuropediatrics, Charité-Universitätsmedizin Berlin, Berlin, Germany
* kerim.mutig@charite.de
§ These authors have contributed to the study equally.

Original immunoblot scans

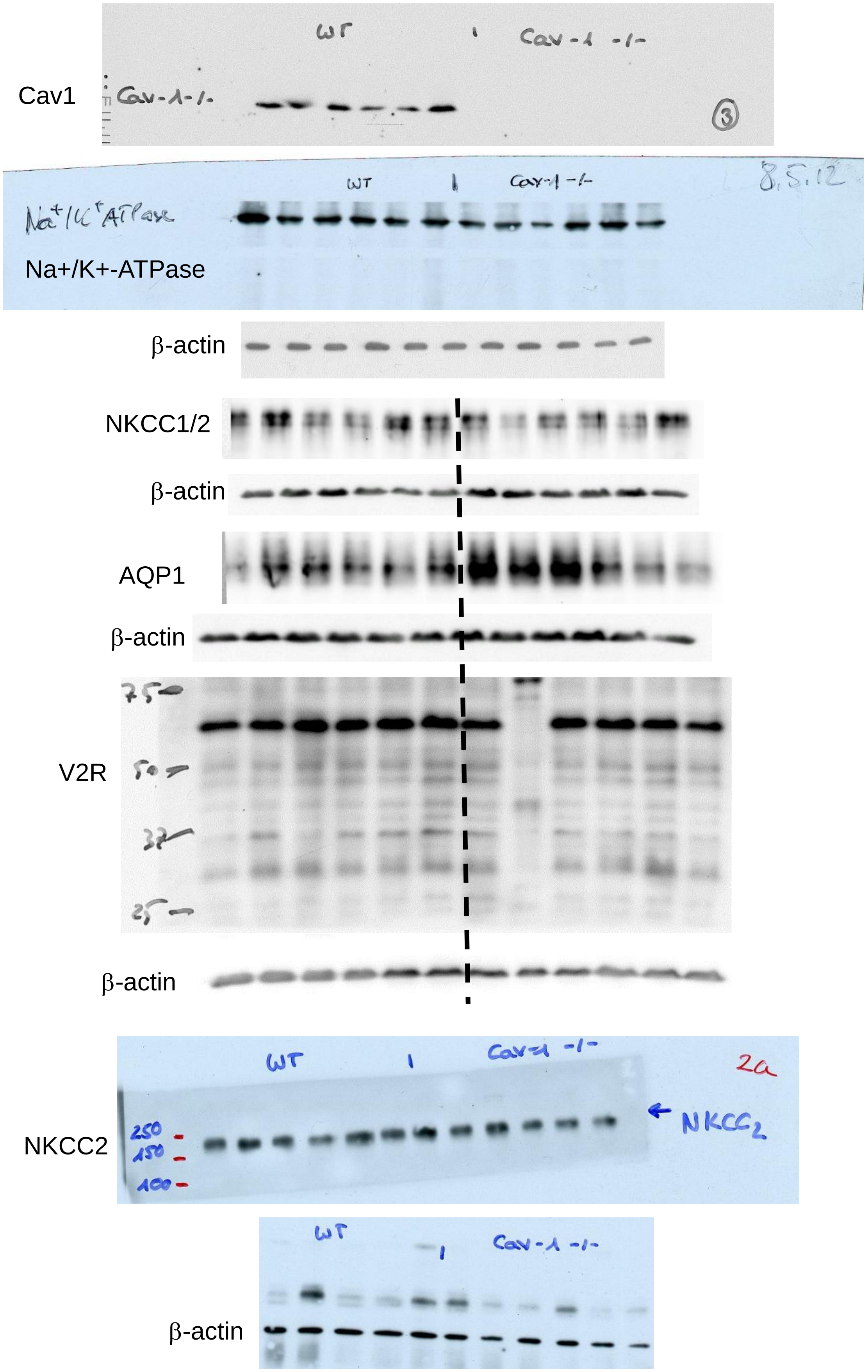

pNKCC2

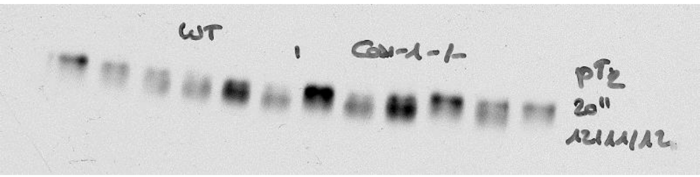

β-actin

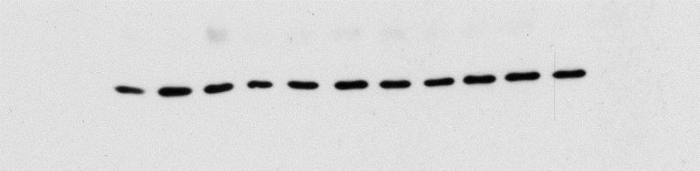

NCC

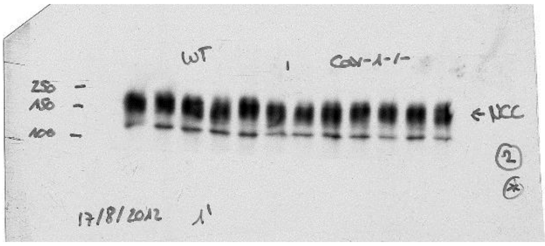

β-actin

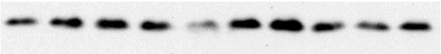

pNCC

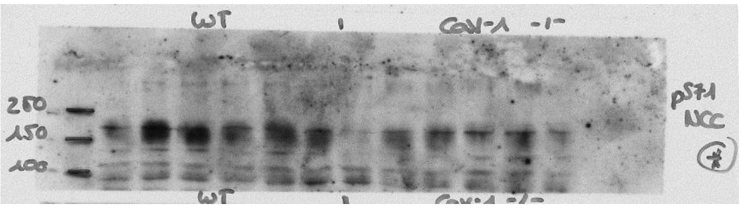

β-actin

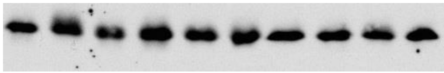

AQP2

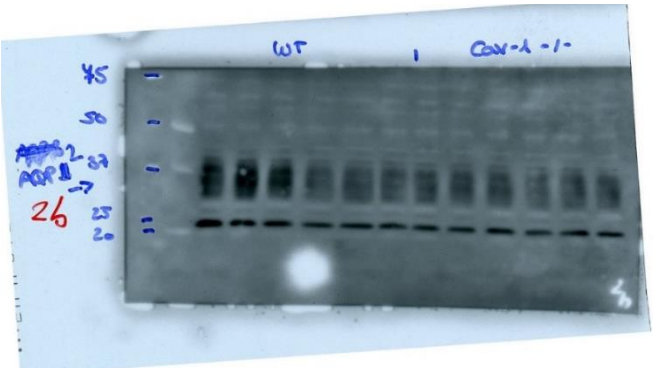

β-actin

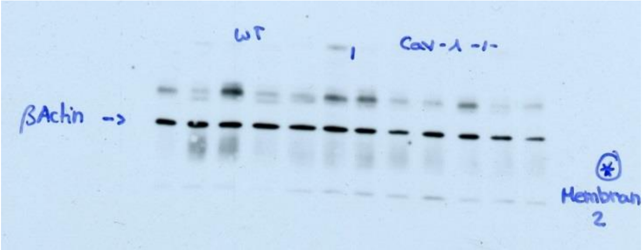

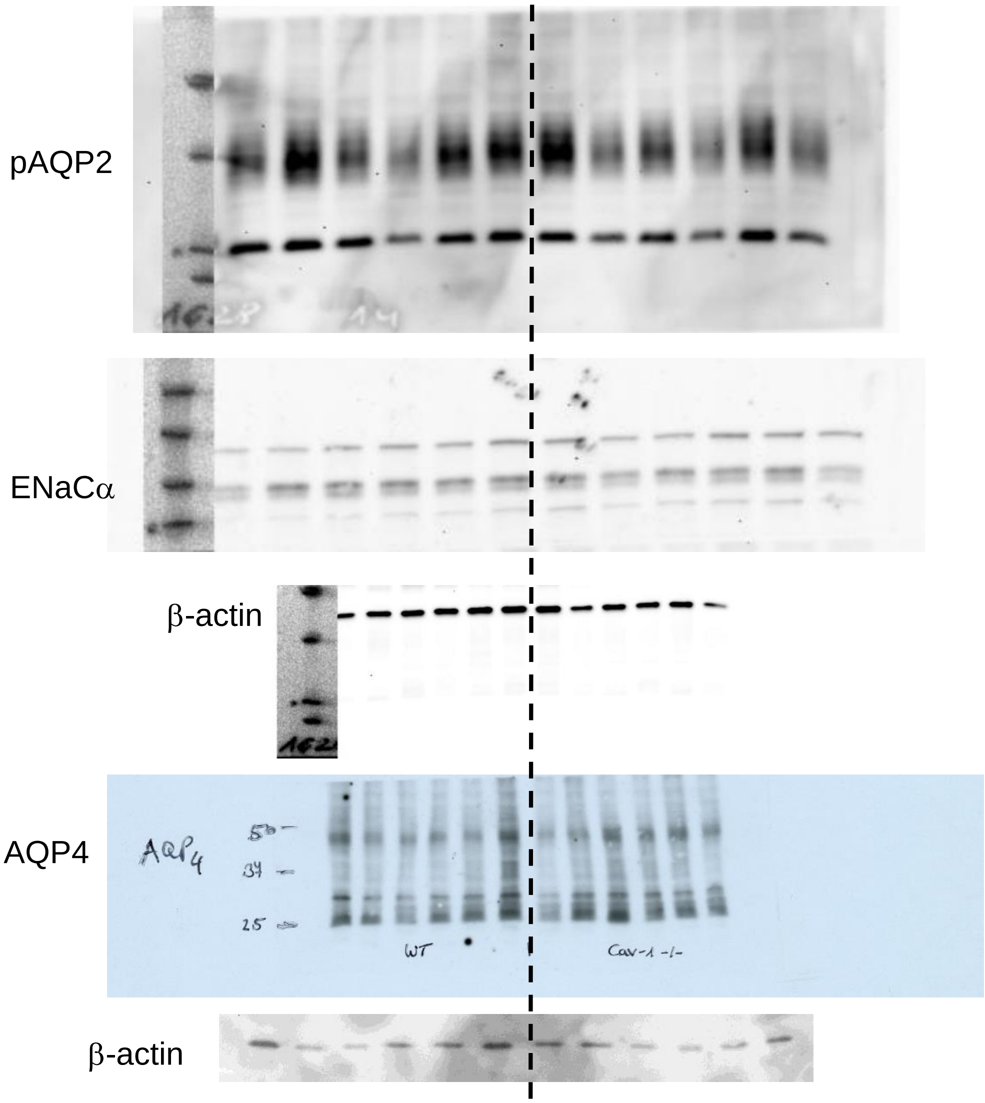
pAQP2
ENaCα
β-actin
AQP4
β-actin

Original immunoblot scans for Cav1, alpha subunit of Na/K-ATPase (Na^+/K^+), NKCC1/2 (antibody recognizes both NKCC isoforms), AQP1, V2R, NKCC2, phosphorylated (p) NKCC2 (pT96/T101-NKCC2), NCC, pNCC (pS71-NCC), AQP2, pAQP2 (pS256-AQP2), alpha subunit of epithelial sodium channel (ENaCα) and aquaporin 4 (AQP4); pAQP2 and ENaCα were detected on the same membrane cut according to their molecular sizes and therefore share the same loading control; loading controls via β-actin detection are shown below the corresponding immunoblots.
